# A novel index of infarct morphology predicts the presence of microvascular obstruction in patients with acute myocardial infarction

**DOI:** 10.1186/1532-429X-14-S1-P23

**Published:** 2012-02-01

**Authors:** Lowie M Van Assche, Han W Kim, Sebastiaan C Bekkers, Brenda Hayes, Michele Parker, Raymond J Kim

**Affiliations:** 1 Cardiology, Duke University, Durham, NC, USA; 2 Cardiology, Maastricht University, Maastricht, Netherlands

## Background

Microvascular obstruction (MO) has been associated with poor LV remodeling and adverse prognosis. Infarct morphology is related to the presence of MO in that patients with MO generally have larger infarct size (IS) and greater mean infarct transmurality. However, neither index is highly predictive on an individual patient basis. In the current study, we investigated the utility of a novel index of infarct morphology, which reflects the circumferential extent of fully transmural infarction extending to the epicardial surface−the epicardial surface area (EpiSA) of infarction−to predict MO.

## Methods

We studied 302 consecutive patients from 2 centers (Duke and Maastricht University) with first AMI. On contrast-enhanced-CMR, early (2-min post-contrast) and late MO (10-min post-contrast) were defined as hypoenhanced regions within hyperenhanced infarction. Infarct size, mean transmurality, and EpiSA were quantified by manual planimetry of the stack of short-axis views.

## Results

**Figure 1:**
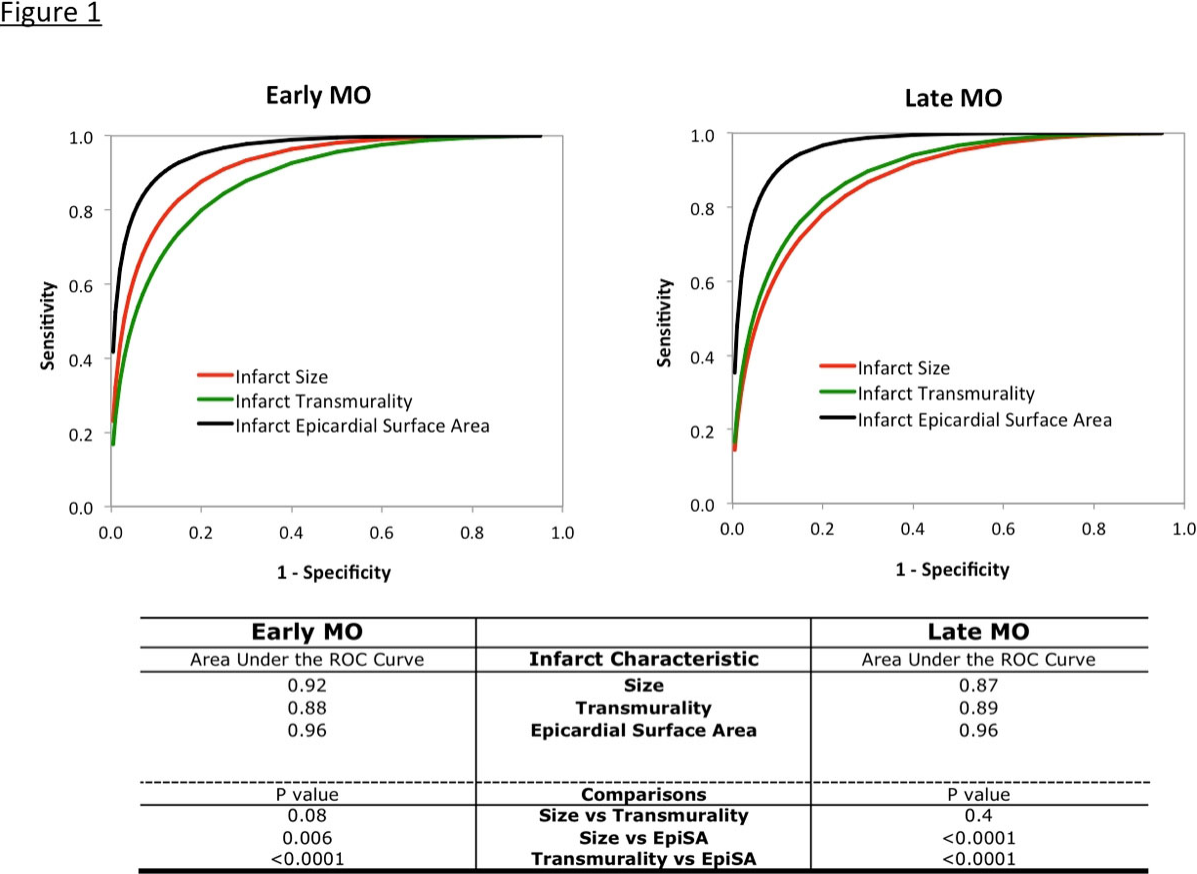


Patients were 58±11 years old (71% male). Prevalence of early and late MO was 64% and 55%, respectively. For the population, IS, mean transmurality, and EpiSA were 14% of LV mass (IQR 7-25%), 74% of infarct sector (IQR 57-86%) and 6% of total LV epicardial-surface-area (IQR 1-13%), respectively. All 3 infarct characteristics were significantly larger in patients with MO (all p<0.0001). On ROC-curve analyses, EpiSA predicted MO more accurately (e.g. larger area-under-the-curve) then IS or mean transmurality (Figure 1). For the 3 infarct characteristics, Table 1 shows threshold, cut-off values for which MO was always absent or always present in the population. For instance, MO (early or late) was always absent when infarct size was <1.4% of LV (5% of population), and always present when >42% of LV (4% of population). However, only a small portion of the population (5%+4%=9%) had infarct size reaching these thresholds, showing that IS had limited discriminatory value on an individual patient basis. Similarly, infarct transmurality had limited discriminatory value. In contrast, EpiSA thresholds allowed ruling-in or ruling-out MO in a significantly larger percentage of the population (44% for both early and late; p<0.0001 compared with IS and transmurality). No patient had MO unless EpiSA was greater than zero. Multivariable analysis incorporating clinical, ECG, and CMR data demonstrated that EpiSA was the strongest, independent predictor of early and late MO (p<0.0001 for both).

**Table 1:** Presence or absence of MO according to thresholds of infarct characteristics

	Microvascular obstruction		
	
Early MO	Always absent	Absent or present	Always present
**Infarct size**			
% LV threshold	<1.4	1.4 – 42	>42
% population represented	5%	92%	4%
**Infarct transmurality**			
% LV threshold	<35	35 – 95	>95
% population represented	4%	89%	7%
**Infarct epicardial surface area**			
% total LV surface area threshold	<0.3	0.3 – 13.6	>13.7
% population represented	20%*	56%	24%*

Late MO			

**Infarct size**			
% LV threshold	<1.4	1.4 – 42	>42
% population represented	5%	92%	4%
**Infarct transmurality**			
% LV threshold	<35	34 – 98	>99
% population represented	4%	95%	1%
**Infarct epicardial surface area**			
% total LV surface area threshold	<0.3	0.3 – 13.6	>13.7
% population represented	21%*	56%	23%*

* % population represented is greater than corresponding population for infarct size and transmurality (p<0.0001).

## Conclusions

The epicardial surface area of infarction, a novel index of infarct morphology, is a stronger predictor of MO than infarct size or mean transmurality. MO does not occur unless infarction extends to the epicardial surface.

## Funding

Funded in part by 5R01HL064726-07.

